# Study on the influence of different production factors on PSY and its correlation

**DOI:** 10.1186/s40813-022-00253-2

**Published:** 2022-03-14

**Authors:** Ran Guan, Xingdong Zhou, Hongbo Cai, Xiaorui Qian, Xiaoyu Xin, Xiaowen Li

**Affiliations:** 1 Shandong New Hope Liuhe Agriculture and Animal Husbandry Technology Co., Ltd (NHKH Academy of Swine Research), No. 6596 Dongfanghong East Road, Yuanqiao Town, Dezhou, 253000 Shandong People’s Republic of China; 2 Key Laboratory of Applied Technology on Green-Eco-Healthy Animal Husbandry of Zhejiang Province, Zhejiang Provincial Engineering Laboratory for Animal Health Inspection & Internet Technology, Zhejiang International Science and Technology Cooperation Base for Veterinary Medicine and Health Management, China-Australia Joint Laboratory for Animal Health Big Data Analytics, College of Animal Science and Technology & College of Veterinary Medicine of Zhejiang Agriculture and Forestry University, 666 Wusu Street, Lin’an District, Hangzhou, 311300 Zhejiang People’s Republic of China; 3 Intelligent Engine Department, The Ant Financial (Hang Zhou), Network Technology Co., Ltd, A Space, No. 569 Xixi Road, Hangzhou, People’s Republic of China; 4 Gansu New Hope Liuhe Agriculture and Animal Husbandry Co., Ltd, No. 1 Zhongchuan Street, Lanzhou New Area, Yongdeng County, Lanzhou, 730087 Gansu People’s Republic of China

**Keywords:** PSY, Correlation coefficient, Mating rate within 7 days after weaning, Farrowing rate, Number of piglets born alive per litter, Number of weaned piglets per litter

## Abstract

**Background:**

Finding out the key reproductive performance factors, affecting piglets weaned per sow per year (PSY) can improve the production efficiency and profitability of pig farms. The objective was to understand the actual distribution of different production factors and PSY of breeding pig farms, analyze the correlation to find the main production factors affecting PSY, and formulating a Production Efficiency Improvement Plan in practice. Data included 603 breeding pig farms from September 28, 2020 to September 26, 2021. Regression analysis was used to evaluate the relationship between PSY and key production factors, and the characteristics of total pig farms versus high performance (HP) pig farms (the production performance was in the top 10%) or top 5% pig farms were compared. Spearman’s rank correlation coefficient was used to analyze the correlation between production factors and find the factors related to PSY. Non-linear support vector regression (NL-SVR) was used to analyze the personalized PSY improvement through a various change of the four key factors.

**Results:**

The median distribution of 15 production factors and PSY in total pig farms were different from those of HP farms. All of data were distributed nonlinearly. Mating rate within 7 days after weaning (MR7DW), farrowing rate (FR), number of piglets born alive per litter (PBAL) and number of weaned piglets per litter (WPL) were moderately correlated with PSY, and the correlation coefficients were 0.5058, 0.4427, 0.3929 and 0.3839, respectively. When the four factors in NL-SVR changed in medium (0.5 piglet or 5%) or high level (1.0 piglet or 10%), PSY can be increased by more than 0.5.

**Conclusion:**

NL-SVR model can be used to analyze the impact of changes in key production factors on PSY. By taking measures to improve MR7DW, FR, PBAL and WPL, it may effectively improve the current PSY and fully develop the reproductive potential of sows.

## Background

Piglets weaned per sow per year (PSY) is an important factor to measure the efficiency of pig farms and the reproductive performance of sows. It is closely related to number of weaned piglets per litter, farrowing rate, non-productive days (NPD) and other production factors [1]. By increasing PSY, the purchase cost of gilts and the feeding cost of sows can be shared equally among more weaned piglets, to improve the profits of commercial pig farms [2]. PSY has been used to provide target for the reproductive performance and productivity of breeding herds [3].

Pig farms management based on productive data analysis can help producers and veterinarians maximize the lifetime reproductive potential of sows and improve economic efficiency [4, 5]. In China, large-scale pig farms are using data management systems to record production data every day [6]. However, most producers only use basic data for current production arrangements. Researchers mainly use linear models such as mixed effect model [7], general linear model [8] and multiple linear regression model [9] for further analysis of production data, which has certain limitations when making accurate calculation or prediction for nonlinear factors. Therefore, this study intends to carry out personalized PSY prediction of each pig farm through non-linear support vector regression model (NL-SVR) and provide scientific reference for production management to formulate targeted production objectives by counting the change distribution of PSY under three improvement levels of different production factors.

## Methods

### Farm description

The study did not require approval from the Ethics Committee on Animal Use because no animal was handled. This was a cross-sectional study involving samples of 603 pig breeding farms from 144 large-scale breeding companies. They fulfilled the following inclusion criteria, which were (1) having a population of 1000 or more sows, (2) using the internal data management system of the company, (3) complete data records. In addition, all pig farms were two-point breeding farms, and weaned piglets were transferred to special commodity farms for feeding and slaughtering. The replacement gilts in estrus or sows after farrowing were fed in the stalls from checking estrus, breeding to late gestation (generally three days before farrowing).

The farms were from 22 provinces, located in the various regions of the country, namely, the East China (33.0%), North China (17.9%), South China (14.3%), Central China (9.0%), Northwest (9.1%), Northeast (8.3%) and Southwest (8.5%) regions.

All these farms applied automatic feeding system (the feed was transferred from galvanized sheet silo to stainless steel feeders (gilts) or DL6 (a commercial model of feeder, which was suitable for 60 chain-disc feed line, with transparent feed doser and fixed throat band, and the maximum feed storage capacity was 6 L. Manual or electric feed drop can be realized, and the volume in the feed doser can be quantified by adjusting the scale.) feed doser (sows) through auger feed line controlled by feed line controller.) and mechanical ventilation system (climate controller for controlling fans of different size). At different growth stages, pigs were fed with the corresponding formula of standardized feed (according to the reference feeding amount, gilts and sows were fed the corresponding 12 kinds of feeds in the stages of nursery, growth, fattening, pregnancy and lactation) provided by the company's internal feed factory. All farms used artificial insemination to mate gilts and sows, and two or three inseminations were carried out in each estrus cycle.

### Data collection and manipulation

The production data were uploaded to the internal data management system by each pig farm. All data belonged to the company. The researchers were authorized by the company's production management department and digital technology department to obtain the production data in this study. This study analyzed 603 large-scale (1000–3000 sows) pig farms from September 2020 to September 2021. Because this study was based on the statistical analysis of pig farms and the amount of data was relatively small, in order to ensure the basic operation of the algorithm model, there was no excessive processing of the original data.

Firstly, the trend relationship between 15 production factors and normalized PSY in 603 pig farms from management reports was analyzed. The calculation method of normalized PSY was as follow:

$$y_{i} = \frac{{X_{i} - \min \{ X_{j} \} }}{{\max \{ X_{j} \} - \min \{ X_{j} \} }}$$

where *X*_*i*_ means the actual PSY for *i* farm; X*j* means a vector consisting of all variables of the number *j* farms, *y*_*i*_ $\epsilon$ [0%, 100%].

Secondly, spearman's rank correlation coefficient was used to analyze the correlation among 16 production factors (including PSY), so as to find the factors related to PSY. According to the data distribution trend, the NL-SVR was selected to analyze the personalized impact of the changes of four production factors with the highest correlation on PSY. Mating rate within 7 days after weaning (MR7DW) and farrowing rate (FR) were set at three levels: high (10%), medium (5%) and low (1%), while number of piglets born alive per litter (PBAL) and number of weaned piglets per litter (WPL) were set at three levels: high (1 piglet), medium (0.5 piglet) and low (0.1 piglet). The distribution of the number of farms corresponding to the change of PSY under different levels of production factors improvement was counted respectively.

### Definitions and categories

Research stage was defined as the stage from September 2020 to September 2021. Total number of piglets was defined as the sum of the total number of piglets sows farrowed during research stage. The NPDs referred to other days except the production days, including mating to pregnancy loss, pregnancy loss to return-service, pregnancy loss to present/culling, weaning-mating, weaning to present/culling. Other definitions were shown in Table 1. The production performance which was in the top 10% referred to high performance (HP) pig farms.

**Table 1:** Descriptions of productive performance between total pig farms and top 5% pig farms

	Mean	Median	Minimum	Maximum	SD
	Total pig farms (n = 603)/Top 5% pig farms (n = 29)				
Total number of piglets per litter^a^	11.3/12.1	11.4/12.2	9.0/10.0	14.9/14.9	1.0/0.9
Number of piglets born alive per litter^b^	10.4/11.5	10.4/11.5	7.5/9.4	14.1/14.1	1.0/0.9
Number of weaned piglets per litter^c^	9.4/10.5	9.4/10.4	6.8/9.0	12.4/12.4	1.0/0.6
Farrowing rate (%)^d^	78.2/87.4	81.2/88.5	36.6/58.5	99.2/99.2	12.3/6.8
Stillbirth rate (%)^e^	6.4/3.9	5.7/3.6	3.1/0.6	18.4/8.8	2.9/1.7
Mummified piglets rate (%)^f^	2.0/1.4	1.7/1.3	0.6/0.0	9.9/5.2	1.2/0.9
Return-service rate (%)^g^	14.4/6.0	12.9/5.0	2.7/1.2	46.9/13.0	8.3/3.5
Mating rate within 7 days after weaning (%)^h^	55.7/69.2	58.2/69.9	0.0/19.2	85.8/83.8	16.5/12.6
Weaning to breeding interval^i^	17.2/9.6	12.8/9.0	4.7/6.5	23.5/22.0	13.8/3.1
Non-productive days^j^	84.1/50.0	76.8/45.7	27.4/27.7	270.0/113.6	38.2/17.8
Production days^k^	686.5/1481.0	502.0/763.0	120.0/280.0	5176.0/5176.0	768.6/1368.1
Birth weight of piglets (kg)^l^	1.2/1.2	1.2/1.2	1.0/1.0	1.6/1.5	0.1/0.1
21-day adjusted weight of piglets (kg)^m^	5.7/6.0	5.7/6.2	4.4/4.9	7.3/7.3	0.5/0.5
Longitude	113.8/111.4	113.5/109.0	104.8/103.2	128.8/123.4	5.6/7.1
Latitude	33.9/33.8	35.1/34.1	23.3/23.4	47.7/42.7	6.0/5.3

^a^Total number of piglets per litter: Total number of piglets per litter/Number of litters

^b^Number of piglets born alive per litter: Number of piglets born alive/Number of litters

^c^Number of weaned piglets per litter: Number of weaned piglets/Number of litters

^d^Farrowing rate: Number of farrowed litters/(Number of farrowed litters+Gestation loss of 115 days pushed forward in the research stage)

^e^Stillbirth rate: Number of stillbirth/Total number of piglets

^f^Mummified piglets rate: Number of mummified piglets/Total number of piglets

^g^Return-service rate: Number of return service sows/Number of mating sows

^h^Mating rate within 7 days after weaning: Number of mating sows within 7 days after weaning/(Number of weaned sows7 days ago—number of culling sows 3 days ago—number of dead sows within 7 days after weaning)

^i^Weaning to breeding interval: The first mating date of the sow in this breeding cycle—The weaning date of the sow in the same parity

^j^Non-productive days: (Sum of non-productive days of all sows in the research stage/Days in the research stage) × 365.25/Average number of sows in the research stage

^k^Production days: Date of the last day of the research stage—Date of production started

^l^Birth weight of piglets: Sum of birth litter weights in all birth records during the research stage/Number of born alive piglets

^m^21-day adjusted weight of piglets: Actual average weaning weight×(2.218−0.0811×Average weaning age+0.0011×Average weaning age^2)

### Statistics analysis

All analyses were conducted with python programming language in PyCharm CE. The farm was considered the experimental unit. In order to reduce the noise in raw data, abnormal data points were deleted, such as the PSY of zero, the farrowing rate of zero, the average number of piglets born alive per litter of zero.

Spearman's rank correlation analysis between 16 factors (including PSY) was performed to construct the correlation coefficient matrix. The correlation between variables and PSY, and the collinearity between each variable was analyzed by this analysis.

NL-SVR model in the sklearn algorithm library was used to learn the data of more than 600 pig farms, obtaining the model after fitting and convergence. After a variable in the data was increased by delta, a prediction data set was generated. Followed by the data set prediction with the model, the change of PSY of each pig farm after the variable was increased by delta was obtained. The kernel function is radial basis function (RBF).

## Results

**Fig. 1:**
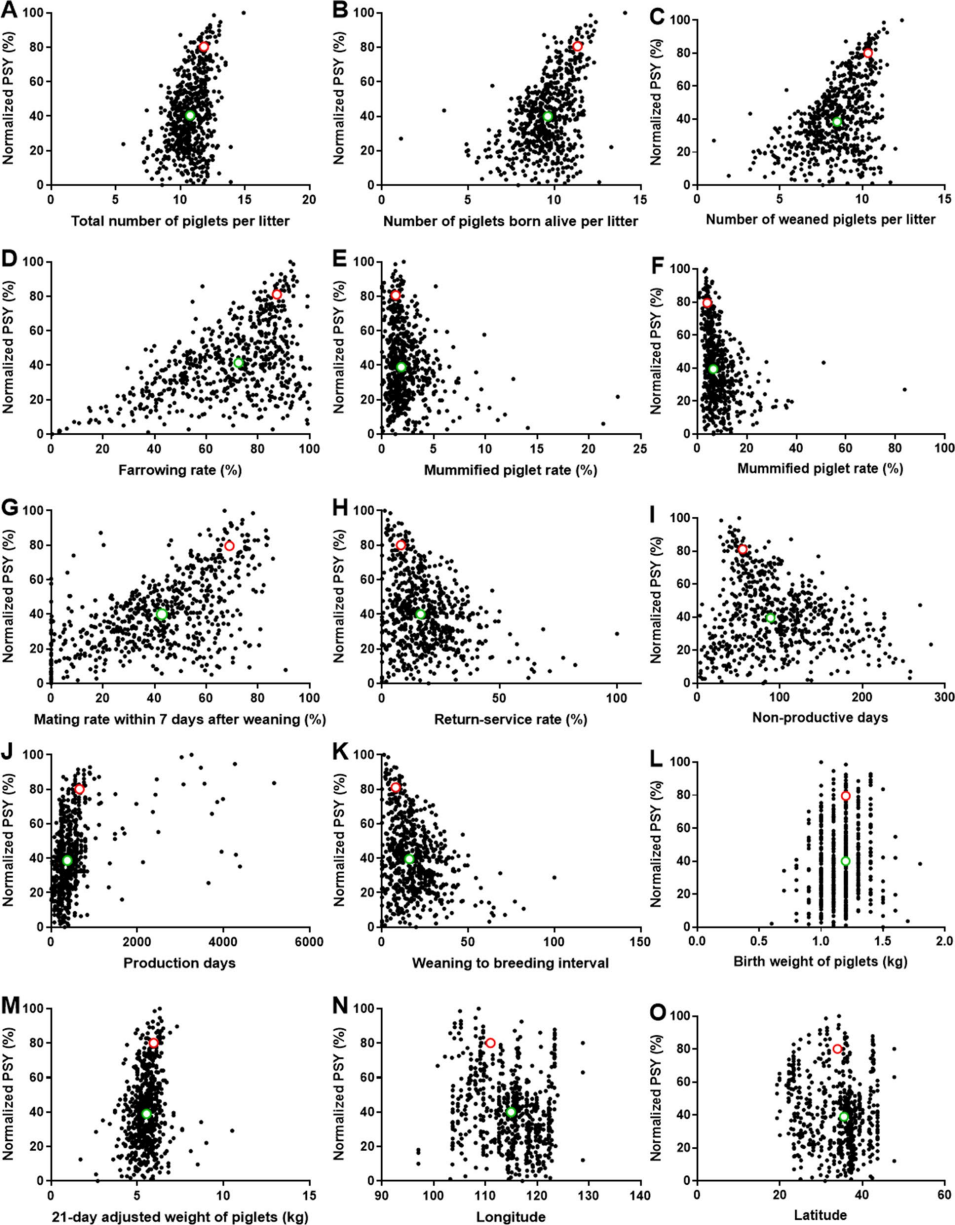
Relationship between 15 production factors and PSY in 603 farms. The green dot represents the median of the factor and PSY in all farms (n=603), and the red dot represents the median of the factor and PSY in high performance pig farms (the production performance is in the top 10%, n=60)

A total of 16 production factors in 603 pig farms were analyzed. The relationship between 15 production factors and normalized PSY (%) in all farms was shown in Fig. 1. The median of factor versus PSY from all pig farms (green dot) was visually lower than that of HP farms (red dot), which was distributed in the left (Fig. 1A–D, G, J, M), right (Fig. 1E, F, H, I, K, N, O) or middle (Fig. 1L) of the red dot according to different factors. The specific statistical data were shown in Table 1. The mean, median, minimum and maximum of PSY and positive factors (such as total number of piglets per litter (TPL), PBAL, WPL, FR, MR7DW, production days, and 21-day adjusted weight of piglets in HP farms were higher than that of total pig farms. The mean and median of birth weight of piglets were equal, while the minimum and maximum were still higher. Negatively related factors, such as stillbirth rate (SR), mummified piglets rate, return-service rate, weaning to breeding interval (WBI) and NPDs in HP farms were lower.

**Fig. 2:**
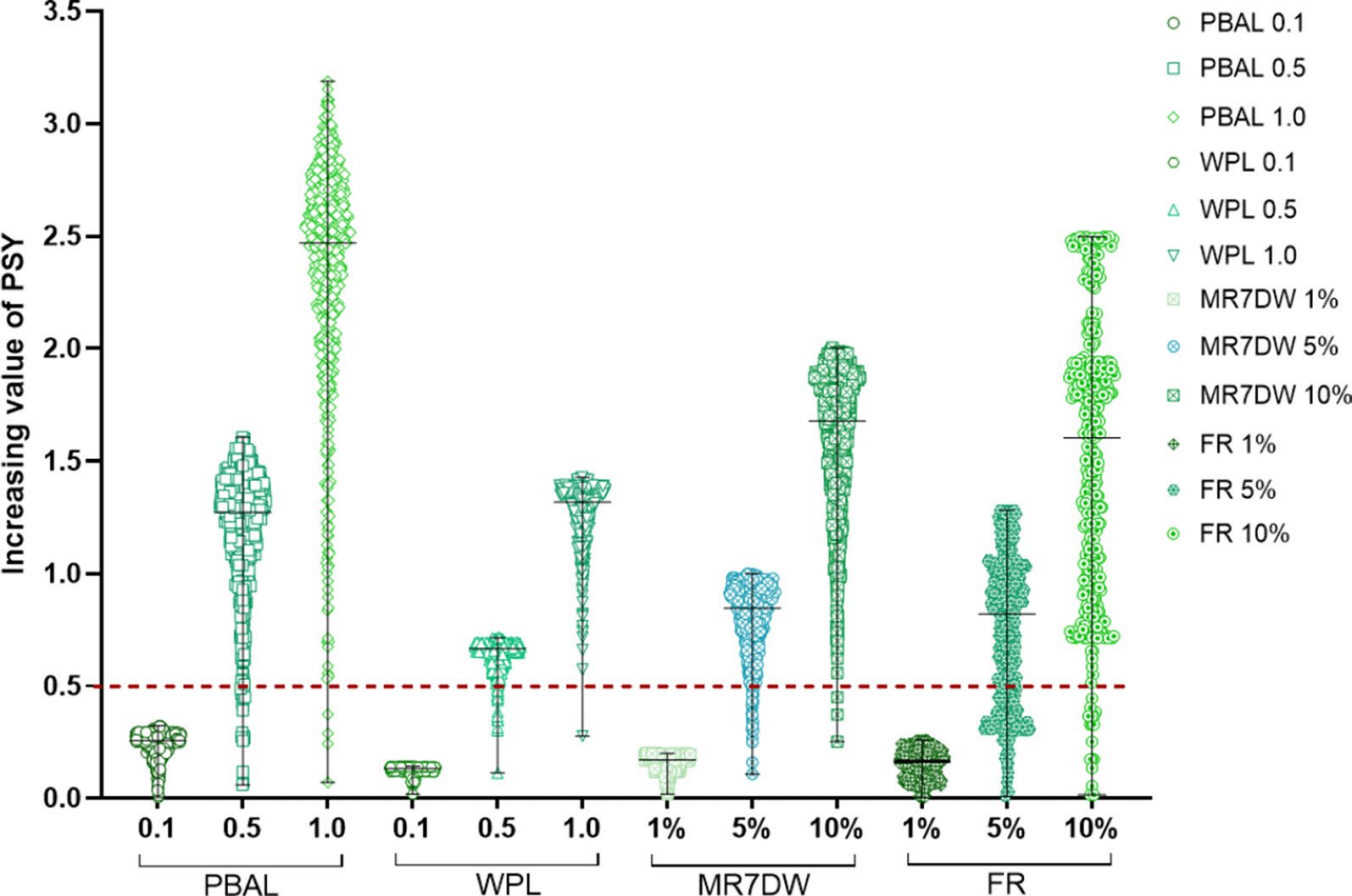
The improvement of production factors corresponds to the change of PSY and the distribution of pig farms. Each factor is represented by a geometric graph, and the width of graph clustering represents the degree of data concentration. PBAL and WPL were divided into three promotion levels, 0.1 piglet (low level), 0.5 piglets (medium level) and 1.0 piglet (high level), respectively. While MR7DW and FR were divided by 1% (low level), 5% (medium level) and 10% (high level), respectively. The red dotted line represents the distribution of other factors when PSY increases by 0.5. PBAL, number of piglets born alive per litter; WPL, number of weaned piglets per litter; MR7DW, mating rate within 7 days after weaning; FR, farrowing rate

The correlation coefficient matrix of 16 production factors in 603 farms were shown in Fig. 2. PBAL versus TPL represented the highest correlation (0.8916), followed by PBAL versus WPL (0.8487). The factor with the highest correlation with PSY was MR7DW (0.5058), followed by FR (0.4427), PBAL (0.3929) and WPL (0.3839), respectively. The top three factors related to MR7DW were PBAL (0.5978), WPL (0.5780) and WBI (−0.4922), respectively. The top three factors related to FR were NPD (−0.6346), WPL (0.4052) and PBAL (0.3748), respectively. The another top two factors related to WPL were TPL (0.7115) and SR (−0.5879), respectively.

Figure 2 showed the distribution of pig farms and corresponding PSY when the four factors were raised at different levels. With the increase of the levels, PSY increased in varying degrees, but the low-level improvement could not increase 0.5 PSY. Among them, PBAL had great promotion potential. When one PBAL was promoted, average PSY can improve nearly 2.5.

## Discussion

Sows have the potential to produce about 60–70 weaned piglets per life [10]. If the annual parities were calculated as 2.27, the average PSY should reach more than 26. However, our data showed that WPL was only 9.4 and the HP farm was 10.5 (Table 1), which indicated that there was a lack of more than two piglets per sow per year, so this was an opportunity to improve farm level productivity. Our results also showed that improving MR7DW, FR, PBAL or WPL can effectively improve the overall PSY of the pig farms (Fig. 2). This was similar with the results of Tani’s study [7].

The average of all sows was calculated in each pig farm within one year (September 2020–September 2021), eliminating the seasonal effect of the observed response [8]. Through scatter plot of data analysis (Fig. 1), we found that the relationship between each variable and PSY was not typical linear but determined the upper boundary of PSY. The higher the dispersion of data, the greater the randomness. Using the general linear models can only get a general trend, but there may be a large deviation from the actual situation. Therefore, instead of linear regression, the NL-SVR model was selected. NL-SVR was a technology widely used in the field of data analysis and prediction. The multivariate nonlinear regression method was used to learn by dividing the vector space, obtaining the differentiated results of PSY changes after each field variable was improved (if linear regression was used, only statistical results can be obtained, and individual results of each field cannot be calculated). The advantage of this approach was on learning the nonlinear relationship between variables and targets and calculated the impact on targets when each variable in the sample changes. When applied to the analysis of the relationship between pig production factors, it can analyze the nonlinear relationship between various factors and PSY, calculate the bottleneck of further improvement of PSY in each field, evaluate the difficulty of improvement, optimize the input–output ratio, and realize the improvement of production efficiency. However, by the great variation in management, facilities, sanitation and feedings, many of which will affect the production performance of piglets and sows, or there were differences in calculation standards, resulted in data fluctuations [8, 11].

Among the 15 production factors, the four factors with the highest correlation coefficients with PSY were MR7DW, FR, PBAL or WPL, respectively. Their correlations from each other were also very high (Table 2). Reducing NPD can improve productivity and profitability of pig farms. Because with the increase of NPD, sow maintenance cost increased, and profitability decreased [12]. The cost of each NPD of sows ranged from $ 1.60 to $ 2.60 [13]. NPD was a comprehensive index, which was significantly affected by management factors, including multiple breedings of sows, MR7DW, parity of culled sows, proportion of return-serviced sows, sow mortality, SR and pig farm scale [12, 14]. Increasing MR7DW can shorten NPD, and increasing the lactation period may increase the proportion of estrus in sows within 4–6 days after weaning, which had higher reproductive performance and longer lifetime [15]. However, sows with prolonged lactation will lose a lot of body reserves, which may reduce the farrowing rate [16] and the number of piglets born per sow per year [11], and thus reduce the number of weaned piglets per year [3]. Therefore, it was necessary to optimize feed intake and feeding mode during lactation [16, 17].

**Table 2:**
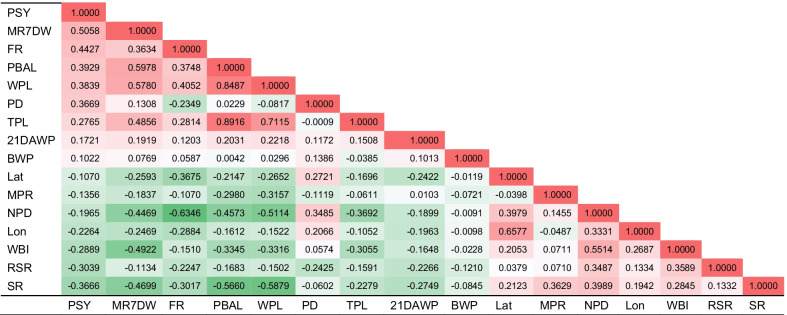
Correlation coefficient matrix of 16 production factors in 603 farms

Red represents positive correlation and green represents negative correlation. The darker the color, the higher the correlation coefficient. PSY, piglets weaned per sow per year; MR7DW, mating rate within 7 days after weaning; FR, farrowing rate; PBAL, number of piglets born alive per litter; WPL, number of weaned piglets per litter; PD, production days; TPL, total number of piglets per litter; 21DAWP, 21-day adjusted weight of piglets; BWP, birth weight of piglets; Lat, latitude; MPR, mummified piglet rate; NPD, non-productive days; WBI, weaning to breeding interval; Lon, longitude; RSR, return-service rate; SR, stillbirth rate

Among the four key production factors, the change of PBAL had the greatest impact on the improvement of PSY. Koketsu et al. [18] found that the average pre-weaning mortality, number of piglets born alive, number of weaned piglets and PSY of herds increased from 2007 to 2016, which may be related to the genetic improvement of pig industry in the past few decades [19, 20]. It was worth noting that the number of weaned piglets didn’t increase continuously with the increased number of piglets born alive. When the number of litters increased from 11–12 to 13–16, the pre-weaning mortality of piglets had almost tripled [21, 22]. Limited by the reproductive capacity of sow itself, larger litter size can lead to reduced piglet birth weight and increased pre-weaning mortality.

**Fig. 3:**
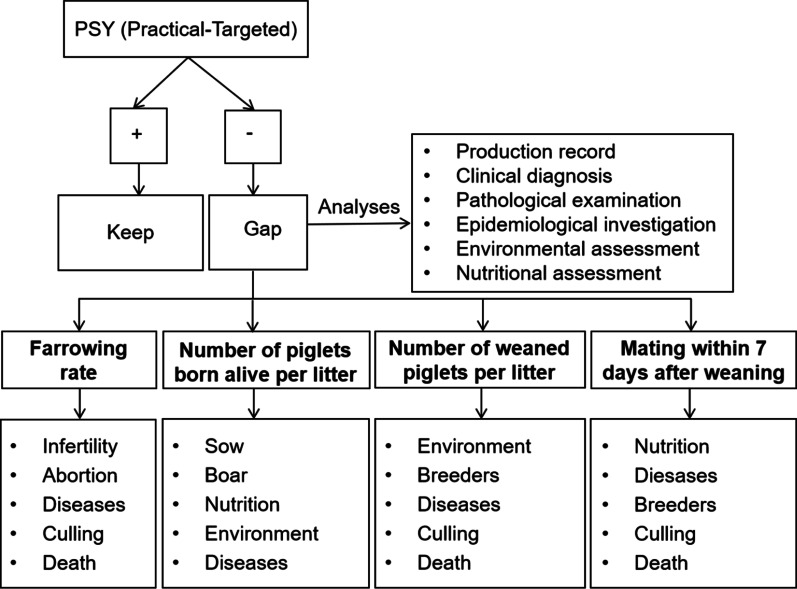
Methods of improving PSY in this study

In order to reduce the waste of production costs and economic benefits on pig farms, it is important for managers to maximize the lifetime reproductive performance of all sows. Through our research, we found four production factors with the highest correlation with PSY. Targeted improvement of these factors may improve the productivity of sows (Fig. 3). In addition, it also needs to be combined with appropriate nutrition [23], feeding pattern [24], development of gilts [25], better breeding management (breeding time, semen quality and stockman skills of breeders) [26, 27], pig health management (control and prevention of infectious and non-infectious diseases) [28, 29], complete buildings (environmental control system, advanced facilities) [30, 31], farrowing management (assisted farrowing, colostrum intake and piglet care) [32, 33] and trained staff [34].

## Conclusions

Our study revealed the nonlinear distribution of production factors with PSY. Among all the production factors analyzed, we found four key factors associated with PSY, which were MR7DW, FR, PBAL and WPL, respectively. The effects of different factors on PSY of each pig farm were analyzed by NL-SVM model, and the distribution statistics were carried out. If targeted improvements were made to the above four factors, especially PBAL, the PSY of pig farms may be improved effectively.

## Data Availability

Due to producer confidentiality, the dataset and farm information are not publicly available.
